# Complexity and Disorder of 1/*f*^α^ Noises

**DOI:** 10.3390/e22101127

**Published:** 2020-10-04

**Authors:** Chang Francis Hsu, Long Hsu, Sien Chi

**Affiliations:** 1 Department of Electrophyics, National Chiao Tung University, Hsinchu 30010, Taiwan; francis-920@hotmail.com (C.F.H.); long@cc.nctu.edu.tw (L.H.); 2 Department of Photonics, National Chiao Tung University, Hsinchu 30010, Taiwan

**Keywords:** 1/*f^α^* noises, power law, complexity, disorder, inverted U curve, heart rate variability, RR interval

## Abstract

The complexity and the disorder of a 1/$f^{\alpha }$ noise time series are quantified by entropy of entropy (EoE) and average entropy (AE), respectively. The resulting EoE vs. AE plot of a series of 1/$f^{\alpha }$ noises of various values of α exhibits a distinct inverted U curve. For the 1/$f^{\alpha }$ noises, we have shown that α decreases monotonically as AE increases, which indicates that α is also a measure of disorder. Furthermore, a 1/$f^{\alpha }$ noise and a cardiac interbeat (RR) interval series are considered equivalent as they have the same AE. Accordingly, we have found that the 1/$f^{\alpha }$ noises for α around 1.5 are equivalent to the RR interval series of healthy subjects. The pink noise at α = 1 is equivalent to atrial fibrillation (AF) RR interval series while the white noise at α = 0 is more disordered than AF RR interval series. These results, based on AE, are different from the previous ones based on spectral analysis. The testing macro-average F-score is 0.93 when classifying the RR interval series of three groups using AE-based α, while it is 0.73 when using spectral-analysis-based α.

## 1. Introduction

It is widely considered that the complexity is relatively high for a complex system intermediate between extreme order and disorder. This results in an inverted U relation between the complexity and the disorder for complex physiologic signals or complex simulated 1/$f^{\alpha }$ noises [1,2,3,4,5,6,7,8,9]. Exploring the resemblance between the two kinds of signals would provide the underlying mechanism of the physiologic signals of health and disease [10,11,12,13,14].

The power spectral density (PSD) of a 1/$f^{\alpha }$ noise is inversely proportional to the frequency *f* to the power of α. The relation between the complexity and the exponent α of 1/$f^{\alpha }$ noises was studied by Zhang [5] and Saito et al [6]. They separately proposed two different measures of complexity for 1/$f^{\alpha }$ noises, which resulting in different inverted U curves of complexity vs. α. However, both methods require more than $10^{6}$ data points to obtain reliable results. It is thus not practical to apply the two measures of complexity to physiological signals of limited data length.

Kobayashi and Musha first found that the PSDs of some RR interval series exhibit a 1/f tendency for frequencies below 0.02 Hz [10]. Later, many other studies found consistent results for the RR interval series of healthy and atrial fibrillation (AF) subjects [11,12,13]. Moreover, some PSDs over higher frequencies were found to be influenced by the autonomic nervous system and breathing activity. Thus, the PSDs of healthy subjects contained fluctuations, [14] while those of the AF patients showed a white noise-like flat spectrum [12,13].

On the other hand, entropy of entropy (EoE) and average entropy (AE) have been shown to be two reliable measures of complexity and disorder of RR interval series with only 500 data points, respectively. EoE is used to differentiate healthy subjects from patients who suffer from heart disease [7]. AE is used to differentiate the three groups of subjects, namely congestive heart failure (CHF), atrial fibrillation (AF), and healthy groups [8]. Furthermore, the expected inverted U curves of EoE vs. AE have been realized for real physiological signals such as heart rate signals and static standing postural stability [7,8,9].

In this paper, we first calculate the EoE and the AE of a series of 1/$f^{\alpha }$ noises with various values of α. We then illustrate a plot of EoE vs. AE and a plot of α vs. AE for the 1/$f^{\alpha }$ noises. For comparison, two similar plots for a series of RR interval series are illustrated. A cardiac RR interval series and a 1/$f^{\alpha }$ noise series are considered equivalent when they have the same AE. We find that the 1/$f^{\alpha }$ noises of different values of α correspond to CHF, AF, and healthy RR interval series, separately.

## 2. Materials and Methods

### 2.1. Simulation of 1/$f^{\alpha }$ Noises

We use the fractional differencing method to simulate 1/$f^{\alpha }$ noises [6,15]. The series of a 1/$f^{\alpha }$ noise $\left\{z_{i}\left(\alpha \right)\right\}$ was obtained by performing the convolution of the uniform white noise $\left\{I_{i}\left(\alpha \right)\right\}$ between −1 and 1 to an impulse response function $\left\{h_{i}\left(\alpha \right)\right\}$, as follows:

(1) $$z_{i}\left(\alpha \right)=\sum_{j=0}^{i}h_{i}\left(\alpha \right)\cdot I_{i-j},$$

where α is the exponent of the 1/$f^{\alpha }$ noise and *i* is the index of the element $z_{i}\left(\alpha \right)$. In addition, the impulse response function $h_{n}\left(\alpha \right)$ is given by

(2) $$h_{i}\left(\alpha \right)=1,\;\left(i=0\right)$$

and

(3) $$h_{i}\left(\alpha \right)=\prod_{k=1}^{i}\frac{\alpha /2+k-1}{k},\;\left(i\geq 0\right)$$

Then, a new series $\left\{x_{i}\left(\alpha \right)\right\}$ was constructed as normalized 1/$f^{\alpha }$ noise as follows:

(4) $$x_{i}\left(\alpha \right)=\frac{z_{i}\left(\alpha \right)-min\left\{z_{i}\left(\alpha \right)\right\}}{max\left\{z_{i}\left(\alpha \right)\right\}-min\left\{z_{i}\left(\alpha \right)\right\}}.$$

In this study, we simulated a series of normalized 1/$f^{\alpha }$ noises, each with a length of 50,000 data points. The α in the series of $\left\{x_{i}\left(\alpha \right)\right\}$ was set to range from 0 to 3 at an interval of Δα=0.01. In total, there are 301 values of α. For each α, 30 simulations of $\left\{x_{i}\left(\alpha \right)\right\}$ were generated. The values of EoE and AE of every $\left\{x_{i}\left(\alpha \right)\right\}$ were evaluated for averages separately. Consequently, a pair of averaged EoE and AE values were obtained for each α. In total, 9030 (=301 × 30) normalized 1/$f^{\alpha }$ noises were analyzed.

### 2.2. Description of Heart Rate Data

The cardiac RR interval time series used in this study were extracted from the following three groups of databases in the online database on Physionet [16], namely (i) the Beth Israel Deaconess Medical Center (BIDMC) Congestive Heart Failure Database (CHFDB), (ii) the Massachusetts Institute of Technology (MIT)–Beth Israel Hospital (BIH) Normal Sinus Rhythm Database (NSRDB), which is a database of healthy subjects, and (iii) the Long Term AF Database (LTAFDB). For convenience, the three databases are abbreviated as CHFDB, NSRDB, and LTAFDB for the purposes of this paper. Note that CHFDB, NSRDB, and LTAFDB originally consist of 15, 18, and 83 24-hour-long electrocardiography (ECG) raw records, respectively. The sampling rate of CHFDB was 250 Hz, while that of the NSRDB and LTAFDB was 128 Hz. Moreover, each of the RR interval series extracted from the long-term ECG raw records contains more than 50,000 data points.

To remove the outlier data points among the RR interval series extracted from the online database, which might be due to noise or detection errors, the following two-step process was applied to each of the long-term RR interval series. First, for each individual data point in an RR interval series, we calculated the mean (MEAN) of the 10 data points before and after the data point itself. The data point under test was removed as it was out of the range MEAN ± 0.5 MEAN. Second, for each of the remaining data points that passed the test in step one, we repeated step one for a new mean (MEAN${}^{\prime }$) and a new standard deviation (SD${}^{\prime }$). The data point under the test was removed as it was out of the range MEAN${}^{\prime }$± 2.5 SD${}^{\prime }$.

Then, for each of the 15 and 18 long-term ECG signals from the CHFDB and the NSRDB, we extracted the first 50,000 data points. As for each of the 83 long-term ECG signals from the LTAFDB, we first extracted all the data segments during AF episodes, according to the annotation in PhysioNet. Among them, we adopted 26 segments whose lengths all exceeded 50,000 data points individually. As a result, there were 59 RR interval series in total, each with 50,000 data points. We then extracted the first 500 or 50,000 data points as short or long time series for the following analysis, respectively.

### 2.3. Entropy of Entropy (EoE) and Average Entropy (AE) Analyses

The algorithms of both EoE [7] and AE [8] methods consist of three steps in analyzing a time series $\left\{x_{i}\right\}=\left\{x_{1},\ldots ,x_{N}\right\}$ of length *N*. The first and the second steps of the two methods are the same for the construction of a Shannon entropy sequence to represent the time series $\left\{x_{i}\right\}$. First, the time series is divided into many consecutive non-overlapping windows of equal length τ. Each window is in the form of $w_{j}^{\left(\tau \right)}=\left\{x_{(j-1)\tau +1},\ldots ,x_{(j-1)\tau +\tau }\right\}$, where *j* is the window index ranging from 1 to N/τ and τ corresponds to the scale factor of EoE and AE.

Second, the Shannon entropy value of each window $w_{j}^{\left(\tau \right)}$ is derived as follows. Suppose that $x_{max}$ and $x_{min}$ are the maximum and minimum of all data collected in this study, respectively. The range from $x_{max}$ to $x_{min}$ is divided into $s_{1}$ slices of equal width $\Delta s_{1}=\left(x_{max}-x_{min}\right)/s_{1}$. The probability $p_{jk}$ for a certain data point xi over window $w_{j}^{\left(\tau \right)}$ to occur in slice *k* is thus obtained in the form of

(5) $$p_{jk}=\frac{\mathrm{total}\;\mathrm{number}\;\mathrm{of}\;\;x_{i}\;\mathrm{over}\;w_{j}^{\left(\tau \right)}\;\mathrm{in}\;\mathrm{slice}\;k}{\tau },$$

where *k* is the state index from 1 to $s_{1}$. Subsequently, the Shannon entropy value $y_{j}^{\tau }$ of each window $w_{j}^{\left(\tau \right)}$ is given by

(6) $$y_{j}^{\left(\tau \right)}=-\sum_{k=1}^{s_{1}}p_{jk}\;\ln\left(\;p_{jk}\right).$$

In this respect, the Shannon entropy value $y_{j}^{\left(\tau \right)}$ is considered the representative of window $w_{j}^{\left(\tau \right)}$. Repeating the same process for every window results in a representative Shannon entropy sequence $\left\{y_{j}^{\left(\tau \right)}\right\}$ of length N/τ for the original time series $\left\{x_{i}\right\}$.

Third, the AE value of $\left\{x_{i}\right\}$ is defined as the average of the Shannon entropy sequence $\left\{y_{j}^{\left(\tau \right)}\right\}$ in the form of

(7) $$AE\left(\tau \right)=\frac{\sum_{j=1}^{N/\tau }y_{j}^{\left(\tau \right)}}{N/\tau }.$$

On the other hand, the EoE value of $\left\{x_{i}\right\}$ is derived as follows. It can be imagined that all elements of $\left\{y_{j}^{\left(\tau \right)}\right\}$ distribute over some finite levels and the maximum number of all possible levels $s_{2}\left(\tau \right)$ depends upon the time scale τ. For example, $s_{2}\left(1\right)=1$, $s_{2}\left(2\right)=2$, $s_{2}\left(3\right)=3$, $s_{2}\left(4\right)=5$, $s_{2}\left(5\right)=7$, and $s_{2}\left(6\right)=11$. Then, the probability $p_{l}$ for a certain representative $y_{j}^{\left(\tau \right)}$ over the sequence $\left\{y_{j}^{\left(\tau \right)}\right\}$ to occur in level *l* is obtained in the form of

(8) $$p_{l}=\frac{\mathrm{total}\;\mathrm{number}\;\mathrm{of}\;y_{j}^{\left(\tau \right)}\;\mathrm{over}\;\left\{y_{j}^{\left(\tau \right)}\right\}\;\mathrm{in}\;\mathrm{level}\;l}{N/\tau },$$

where *l* is the level index ranging from 1 to $s_{2}$. Thus, the EoE value of the original time series $\left\{x_{i}\right\}$ is defined as the Shannon entropy value of the Shannon entropy sequence $\left\{y_{j}^{\left(\tau \right)}\right\}$ and is given by

(9) $$EoE\left(\tau \right)=-\sum_{l=1}^{s_{2}}p_{l}\;\ln\;p_{l}.$$

In this study, $x_{max}=1.6$, $x_{min}=0.3$, τ=14, and $s_{1}=55$ are used for the RR interval series analysis, as suggested in our previous study [8]. Similarly, $x_{max}=1.0$, $x_{min}=0.0$, τ=14, and $s_{1}=55$ are set for the normalized 1/$f^{\alpha }$ noise analysis.

### 2.4. Examples of AE and EoE Analyses of 1/$f^{\alpha }$ Noises

**Figure 1:**
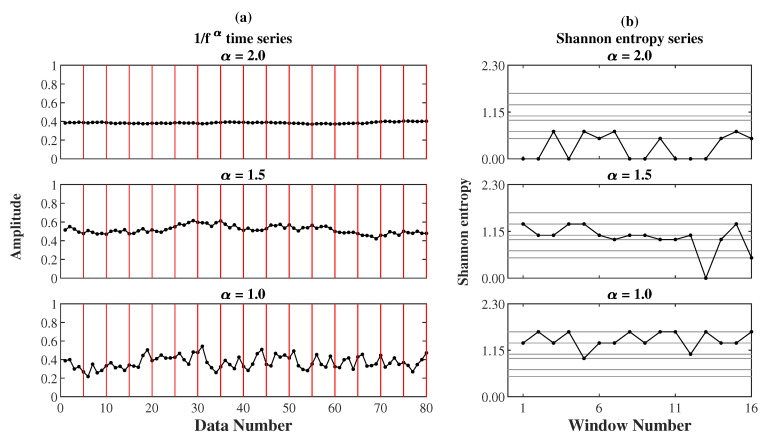
(**a**) The three 1/$f^{\alpha }$ noises $\left\{x_{i}\right\}$ with a same length of 80 data points but different α of 2.0, 1.5, and 1.0, individually. (**b**) The representative Shannon entropy sequences $\left\{y_{j}^{\left(5\right)}\right\}$ of the three 1/$f^{\alpha }$ noises for entropy of entropy (EoE) and average entropy (AE) analyses at τ=5.

Figure 1 demonstrates three 1/$f^{\alpha }$ noises and their representative Shannon entropy sequences for AE and EoE analyses. Figure 1a shows the three 1/$f^{\alpha }$ noises $\left\{x_{i}\right\}$ with the same length N of 80 data points but different α values of 2.0, 1.5, and 1.0 individually. In this case, all 1/$f^{\alpha }$ noises were analyzed at τ=5. It can be seen that the 80 data points of each noise were equally divided into 16(=N/τ) windows with each of the 5 data points in a red frame. Then, the Shannon entropy value of every window in red was calculated individually. Figure 1b shows the representative Shannon entropy sequences $\left\{y_{j}^{\left(5\right)}\right\}$ of the three 1/$f^{\alpha }$ noises separately. Each sequence $\left\{y_{j}^{\left(5\right)}\right\}$ consists of 16 elements separately. According to Equations (9) and (7), the (EoE, AE) values of the three noises were obtained to be (1.04, 0.29), (1.41, 1.00), and (1.07, 1.41), individually. It can be seen that AE increases with increasing α while the maximum EoE occurs at α=1.5.

### 2.5. AE-Based Equivalent 1/$f^{\alpha }$ Noise of an RR Interval Series

In this study, both the AE and the EoE analyses were applied to the 301 simulated 1/$f^{\alpha }$ noises with α from 0 to 3 at Δα=0.01 and the 59 cardiac RR interval time series from 15 CHF, 26 AF, and 18 healthy subjects, separately. We will demonstrate in the results section the resemblance between the 301 1/$f^{\alpha }$ noises and 59 RR interval series in terms of their inverted U curves of EoE (complexity) vs. AE (disorder). This allows us to consider a 1/$f^{\alpha }$ noise and an RR interval series as effectively equivalent as they have the same AE value. Accordingly, each RR interval series can be associated with an equivalent $\alpha_{AE}$. Then, the corresponding 1/$f^{\alpha }$ noise is referred to as the AE-based equivalent 1/$f^{\alpha }$ noise of the RR interval time series.

### 2.6. Spectrum-Based Equivalent 1/$f^{\alpha }$ Noise of an RR Interval Series

The power spectral density of an RR interval time series looks like the power spectral density p(f) of a theoretical 1/$f^{\alpha }$ noise, as given by $p\left(f\right)=c\left(1/f^{\alpha }\right)$, where *c* is a proportional constant. The value of α can be derived from the slope of the $log_{10}\;p\left(f\right)$ vs. $log_{10}\;f$ plot. Consequently, using the least square fitting method, we obtain the optimal value of $\alpha_{sp}$ that yields the best fit for the theoretical power spectral density p(f) to the power spectral density of a measured RR interval time series. The particular 1/$f^{\alpha }$ noise is referred to as the spectrum-based equivalent 1/$f^{\alpha }$ noise of the RR interval time series.

**Figure 2:**
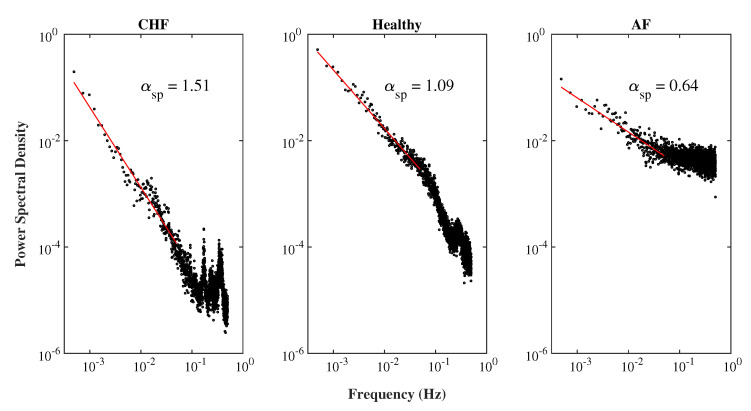
The power spectral densities of the three typical RR interval series from congestive heart failure (CHF), healthy, and atrial fibrillation (AF) subjects in a $log_{10}$ – $log_{10}$ scale, separately. The red line indicates the slope of each power spectral density vs. frequency plot for f≤ 0.02 Hz. The value of each $\alpha_{sp}$ is the product of the slope of the corresponding red line and −1.

In this study, we applied the piecewise cubic Hermite interpolating polynomial method to transform an original RR interval time series vs. beat number into a new RR interval time series vs. time at a sampling frequency of 1 Hz. The power spectral density of the new RR interval series can be obtained by using Welch’s periodogram method for Hamming windows with a window length of 2048 points and an overlap of 50% between consecutive windows. Figure 2 illustrates the power spectral densities of the three typical RR interval series from a CHF, a healthy, and an AF subject in a $log_{10}$ – $log_{10}$ scale, separately. The red line indicates the slope of each power spectral density vs. frequency plot for f≤ 0.02 Hz, as suggested by Kobayashi and Musha [10], which was obtained by using the least square fitting method. The product of the slope of the red line and −1 gives rise to the exponent $\alpha_{sp}$ of the equivalent 1/$f^{\alpha }$ noise of the RR interval series.

### 2.7. Classification of AF, Healthy, and CHF Subjects and Performance Indices

For each RR interval series, we obtain two kinds of equivalent α, namely an AE-based equivalent $\alpha_{AE}$ and a spectrum-based equivalent $\alpha_{sp}$, as stated in Section 2.5 and Section 2.6, respectively.

Quadratic discriminant analysis (QDA) was applied to classify the equivalent α of the RR interval series of the three groups [17]. In the training phase, we calculated the mean and the standard deviation of all the α associated with each group. This allowed the fitting of a Gaussian distribution to the distribution of α in each group. Moreover, three different Gaussian curves, corresponding to the three groups, were obtained. Consequently, the two intersection points of the three Gaussian curves determined the two thresholds for the classification of the equivalent α into three groups.

In the testing phase, we adopted the following three commonly used indices under leave-one-out cross validation (LOOCV) of multi-class classification problems [18]:

(10) $$\mathrm{macro-average}\;\mathrm{recall}:Recall_{M}=\frac{1}{3}\cdot \sum_{i=1}^{3}\frac{TP_{i}}{TP_{i}+FN_{i}}\;,$$

(11) $$\mathrm{macro-average}\;\mathrm{precision}:Precision_{M}=\frac{1}{3}\cdot \sum_{i=1}^{3}\frac{TP_{i}}{TP_{i}+FP_{i}}\;,$$

(12) $$\mathrm{macro-average}\;F\;\mathrm{score}:F_{M}=\frac{2\cdot Recall_{M}\cdot Precision_{M}}{Recall_{M}+Precision_{M}}\;,$$

where *i*, ranging from 1 to 3, indicates one of the three groups of CHF, healthy, or AF patients. $TP_{i}$, $TN_{i}$, $FP_{i}$, and $FN_{i}$ represent true positive, true negative, false positive, and false negative, respectively. Finally, the 95% confidence interval (CI) for $Recall_{M}$, $Precision_{M}$, and $F_{M}$ was obtained by using the bootstrap method with sampling carried 10,000 times.

## 3. Results

**Figure 3:**
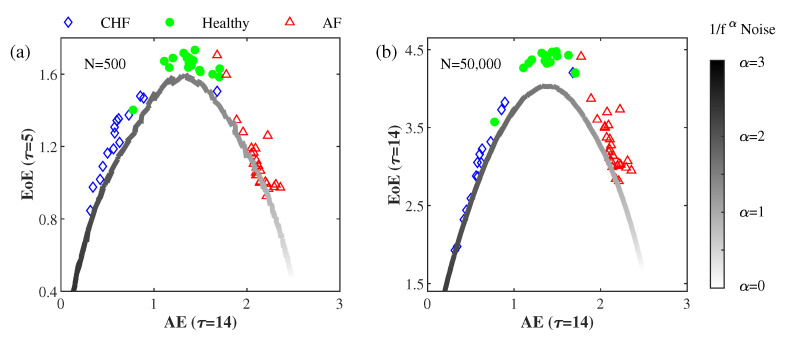
EoE vs. AE plot of the 59 RR interval series and that of the 301 simulated 1/$f^{\alpha }$ noises, each with (**a**) 500 or (**b**) 50,000 data points. The 15 diamonds, the 18 circles, and the 26 triangle symbols are associated with the CHF, healthy, and AF subjects, respectively. The curves in grayscale correspond to the 1/$f^{\alpha }$ noises with α ranging from zero to three at Δα=0.01.

Figure 3a illustrates the EoE EoE (τ = 5) vs. AE AE (τ = 14) of the 15 CHF, the 18 healthy, and the 26 AF sets of short RR interval series (500 data points), as well as those of the simulated 1/$f^{\alpha }$ with α ranging from zero to three at Δα=0.01. Figure 3b illustrates the EoE (τ = 14) vs. AE (τ = 14) of the same data, but with a long data size (50,000 data points). For each α, 30 simulations of normalized 1/$f^{\alpha }$ noises were generated. The values of EoE and AE of every normalized 1/$f^{\alpha }$ noise were separately evaluated to obtain their averages. Consequently, a pair of averaged EoE and AE values was obtained for each α.

From the inverted U curve of the EoE vs. AE plot of the RR interval series, it can be seen that EoE is a good measure of complexity to separate the healthy from the pathologic (CHF and AF). AE is a good measures of disorder to differentiate the three groups of RR interval series. On the other hand, the EoE vs. AE plot of the 1/$f^{\alpha }$ noises exhibits a distinct inverted U curve, which resembles that of the RR interval series but with lower EoE. The EoE value reaches its maximum at α around 1.50, which corresponds to an AE value of 1.45. In this respect, there appears to be a one-to-one relation between AE and α.

**Figure 4:**
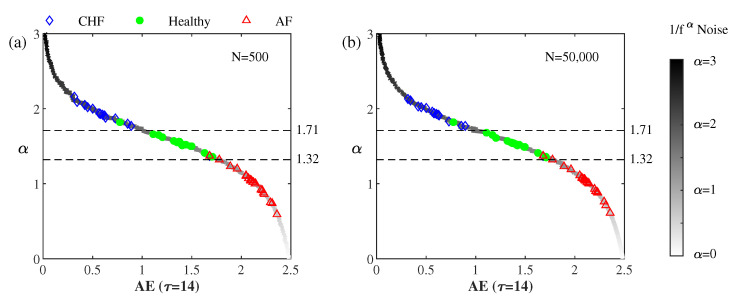
α vs. AE (τ = 14) of the 301 simulated 1/$f^{\alpha }$ noises and the 59 RR interval series for α between 0 to 3 at Δα=0.01. The differentiating thresholds for the classification of the α into CHF, healthy, and AF groups were derived from quadratic discriminant analysis (QDA).

The solid curve in Figure 4 illustrates the relation between AE (τ = 14) and α for the 301 simulated 1/$f^{\alpha }$ noises for α between zero and three at Δα=0.01. It can be seen that α decreases monotonically as AE increases, which implies that α can be a measure of disorder like AE.

An 1/$f^{\alpha }$ noise and an RR interval series are considered equivalent as they have the same AE. Thus, we may assign an AE-based equivalent $\alpha_{AE}$ to an RR interval series. It can be seen that the values of the equivalent $\alpha_{AE}$ of the healthy subjects range between 1.32 and 1.71 while those of the CHF and the AF subjects are larger than 1.71 and smaller than 1.32, respectively.

**Figure 5:**
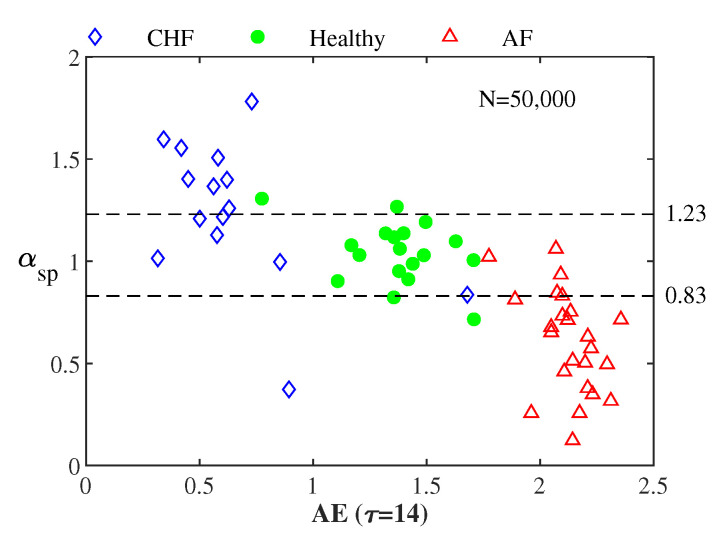
$\alpha_{sp}$ vs. AE for all 59 RR interval series from the 15 CHF, the 18 healthy, and the 26 AF patients.

Figure 5 illustrates $\alpha_{sp}$ vs. AE for all the 59 RR interval series from the CHF, the healthy, and the AF groups. The average $\alpha_{sp}$ values for the 15 CHF, the 18 healthy, and the 26 AF subjects are 1.24, 1.04, and 0.63, respectively. The corresponding $\alpha_{sp}$ of the healthy group was nearly 1.0 in previous studies [10,11,12,13].

Table 1 lists the confusion matrices of LOOCV in differentiating the 59 RR interval series into CHF, healthy, and AF groups using (a) $\alpha_{AE}$ and (b) $\alpha_{sp}$. Note that the differentiated performances using $\alpha_{AE}$ with short and long data sets are the same. Table 2 lists the $Recall_{M}$, $Precision_{M}$, and $F_{M}$ for the same analysis. The 95% confidence interval (CI) for the indices was obtained by using the bootstrap method with sampling carried out 10,000 times. In comparison, the $\alpha_{AE}$ of an RR interval series AE exhibits a better performance.

## 4. Discussion

We have shown that the brown noise at α = 2 resembles the CHF RR interval series. The pink noise at α = 1 resembles the AF RR interval series, while the white noise at α = 0 is more disordered than the AF RR interval series. These results, based on AE, are different from previous studies [10,11,12,13,19].

Previous studies explored the long-range correlation properties [19] of the cardiac RR interval series over the low-frequency domain [10,11,12,13]. Thus, 1/f fluctuations were found in healthy heart rates. However, the analysis requires relatively long data sets to quantify the long-term properties.

On the other hand, AE analysis provides the short-term stability of a short time series. This allows us to explore a reliable correspondence via AE between the simulated 1/$f^{\alpha }$ noises and the real RR interval series.

**Figure 6:**
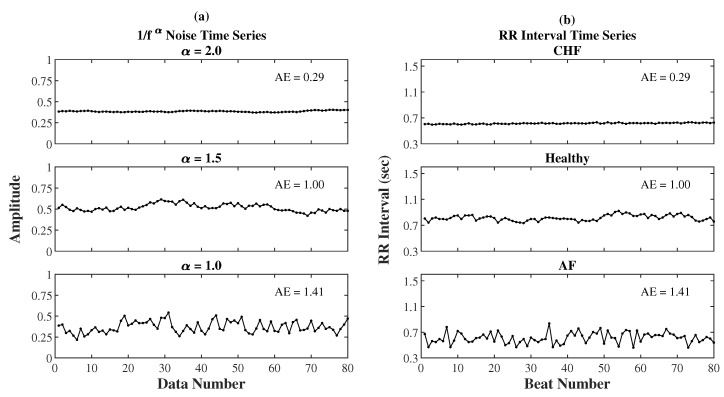
(**a**) Three simulated 1/$f^{\alpha }$ noises with a length of 80 points and α at 2.0, 1.5, and 1.0, separately. (**b**) Three typical RR interval series with a length of 80 points from a CHF, a healthy, and an AF subject, separately. For the three pairs of time series of different α in (a) and (b), the corresponding AE values at τ = 5 are 0.29, 1.00, and 1.41 from top to bottom, respectively.

Figure 6 demonstrates the similarity in the waveforms between three very short RR interval time series and their equivalent 1/$f^{\alpha }$ noises separately. Figure 6a illustrates three simulated 1/$f^{\alpha }$ noises with a same length of 80 points and α at 2.0, 1.5, and 1.0 separately. Figure 6b illustrates three typical RR interval series with a length of 80 points from a CHF, a healthy, and an AF subject separately. For the three pairs of RR interval time series and 1/$f^{\alpha }$ noise of different α in Figure 6a,b, the corresponding AE values at α = 5 are 0.29, 1.00, and 1.41 from top to bottom, respectively. It can be seen that each of the three pairs looks very similar.

## 5. Conclusions

We calculated the EoE and AE of 1/$f^{\alpha }$ noise time series with 50,000 data points for 0≤α≤3. The plot of EoE vs. AE exhibits a distinct inverted U curve. The feature of the inverted U curve remains valid with 80 short data points. We have also shown that α decreases monotonically as AE increases, which indicates that α is also a measure of disorder.

We plotted the EoE vs. AE of 59 cardiac RR interval series, which resembles the inverted U curve of 1/$f^{\alpha }$ noises but with larger EoE. A 1/$f^{\alpha }$ noise and a cardiac RR interval series are considered equivalent as they have the same AE. Accordingly, we found that the 1/$f^{\alpha }$ noise resembles a healthy RR interval series when α is around 1.5. The brown noise at α = 2 resembles the CHF RR interval series. The pink noise at α=1 resembles the AF RR interval series, while the white noise at α = 0 is more disordered than the AF RR interval series. These results, based on AE, are different from the previous ones based on spectral analysis. We have shown that our AE-based results are superior in terms of their accuracy in differentiating between CHF, AF, and healthy groups.

There are two main problems that are worth further exploration. The first problem lies in the clarification of why the equivalent α values of RR interval series obtained from AE and spectral analyses are so different. The second problem is that the EoE values of the 1/$f^{\alpha }$ noises are slightly lower than those of their corresponding AE-based equivalent RR interval series. This implies that differences between the two signals still exist. We believe that exploring a new model for a different simulated signal to fit the cardiac RR interval series better would solve the above two problems.

## Figures and Tables

**Table 1:** Confusion matrices of leave-one-out cross validation (LOOCV) in differentiating the 59 RR interval series into CHF, healthy, and AF groups using (**a**) $\alpha_{AE}$ and (**b**) $\alpha_{sp}$.

	(a) Actual	CHF	Healthy	AF		(b) Actual	CHF	Healthy	AF
Predicted					Predicted				
CHF		14	1	0	CHF		8	2	0
Healthy		11	17	2	Healthy		6	14	4
AF		0	0	24	AF		1	2	22

**Table 2:** $Recall_{M}$, $Precision_{M}$, and $F_{M}$ of LOOCV in differentiating the 59 RR interval series into CHF, healthy, and AF groups using equivalent $\alpha_{AE}$ and $\alpha_{sp}$.

Method	${\mathrm{Recall}}_{M},\%\;95\;\mathrm{CI}$	${\mathrm{Precision}}_{M},\%\;95\;\mathrm{CI}$	${\mathrm{Fscore}}_{M},\%\;95\;\mathrm{CI}$
$\alpha_{AE}$	0.93, [0.86 – 0.99]	0.93, [0.86 – 0.98]	0.93, [0.86 – 0.98]
$\alpha_{sp}$	0.72, [0.57 – 0.81]	0.77, [0.64 – 0.86]	0.73, [0.58 – 0.82]
